# A Case Report of an Unusual Complication from Bee Sting: Acute Brachial Plexopathy

**DOI:** 10.12669/pjms.302.4891

**Published:** 2014

**Authors:** Hoo Fan Kee, Shariful Hasan, Wan Aliaa WS, Hamidon B. Basri

**Affiliations:** 1 Hoo Fan Kee, MD, MRCP (UK), Department of Medicine, Faculty of Medicine and Health Sciences, University Putra Malaysia, Malaysia.; 2 Shariful Hasan, MBBS, FCNp, Department of Medicine, Faculty of Medicine and Health Sciences, University Putra Malaysia, Malaysia.; 3 Wan Aliaa WS, MbBCh BAO LRCP & SI, MRCP (UK), Department of Medicine, Faculty of Medicine and Health Sciences, University Putra Malaysia, Malaysia.; 4 Hamidon B. Basri, MD, MMed, Department of Medicine, Faculty of Medicine and Health Sciences, University Putra Malaysia, Malaysia.

**Keywords:** Anaphylactic shock, Bee sting, Brachial plexopathy, Neurological complication

## Abstract

Brachial plexopathy is usually related to trauma like direct injury to the nerve and stretching injuries. Neurological complications following bee sting are uncommon. Here, we describe a rare case of acute brachial plexopathy as a neurological complication following bee sting. A23-year-old maleinitially presented with angioedema and anaphylactic shock one hour after a bee stung at his neck. Twenty four hours after the incidence, he presented with sudden onset of left upper limb weakness. Nerve conduction study and electromyography had shown evidence of left brachial plexopathy.

## INTRODUCTION

Patients with bee sting usually presented with local reactions and occasionally with systemic allergic reactions such as angioedema or anaphylaxis. Systemic complications that involved nervous system are rare. Stroke, acute polyradiculoneuropathy, trigeminal neuropathic pain, optic neuritis and acute disseminated encephalomyelitis have been reported in association with bee sting.^1^^-^^5^ Incidence of brachial plexopathy or brachial plexitis following bee sting has been rarely described in the literature.^6^^,^^7^

## CASE REPORT

A 23-year-old male presented to us with angioedema and anaphylactic shock approximately one hour after a bee stung at right lower neck. He responded well to intramuscular adrenaline, intravenous hydrocortisone and fluid resuscitation when he arrived at emergency department. Twenty four hours after admission, he developed left upper limb weakness and numbness. He was conscious and hemodynamically stable at this point. He does not have any significant medical history related to this event except a history of bee sting one year ago. Neurological examination of the motor system showed patient was unable to perform left shoulder abduction, flexion and extension (grade 0 /5, according to Medical Research Council Scale for Muscle strength). Similarly, the power of the same side elbow flexion and extension, and left wrist extension were weak (elbow flexion 1/5, elbow and wrist extension 4/5). There were no weakness in left wrist flexion, and fingers extension, flexion, abduction and adduction. Deep tendon reflexes on left biceps and supinator were absent (0/5). The triceps reflex was sluggish (1+). Examination of the sensory system showed sensory deficits (touch, pain, and temperature) in the left C5 and C6 dermatome only. Other clinical examinations of the nervous system were unremarkable. Blood investigations such as full blood count, renal profile, liver function tests and fasting sugar level were all normal. Magnetic resonance imaging (MRI) of cervical spine and brachial plexus were normal. Nerve conduction study and electromyography were consistent with preganglionic left brachial plexus lesion located at the level of C5 and C6 roots. He was discharged with prednisolone 30 mg per day and followed by tapering dose of prednisolone over two weeks. We planned to repeat nerve conduction nerve studies three months later to assess the recovery of C5-C6 brachial plexopathy. 

## DISCUSSION

The pathogenesis of bee sting associated with brachial plexopathy is not fully understood. However, it’s it believed that the nerve damage following a bee sting is probably due to systemic allergic reaction from the bee venom. Bee venoms contain certain potentially allergenic compounds like some peptide toxins and proteins (hyaluronidase, phospholipase A2, etc.).^8^^,^^9^ Hypersensitivity reactions require a pre-sensitised status of the host. In our patient, the previous history of bee sting one year ago may explain the current exaggerated hypersensitivity reaction to the bee venom. Interaction of IgE antibodies with myelin protein resulting from this hypersensitivity reaction is possibly the reason of brachial plexus lesion in our patient. Although MRI of the brachial plexus was normal, nerve conduction studies showed evidence of demyelination in this patient. We conclude that acute brachial plexopathy as a neurological complication following bee sting is possible although it has been rarely reported.

## Authors Contribution:

We certify that we participated sufficiently in the intellectual content, conception and design of this work and the analysis and interpretation of the data (when applicable), as well as writing of the manuscript.
